# Deep analysis of cellular transcriptomes – LongSAGE versus classic MPSS

**DOI:** 10.1186/1471-2164-8-333

**Published:** 2007-09-24

**Authors:** Lawrence Hene, Vattipally B Sreenu, Mai T Vuong, S Hussain I Abidi, Julian K Sutton, Sarah L Rowland-Jones, Simon J Davis, Edward J Evans

**Affiliations:** 1 Nuffield Department of Clinical Medicine and MRC Human Immunology Unit, Weatherall Institute of Molecular Medicine, The University of Oxford, John Radcliffe Hospital, Headington, Oxford, OX3 9DS, UK

## Abstract

**Background:**

Deep transcriptome analysis will underpin a large fraction of post-genomic biology. 'Closed' technologies, such as microarray analysis, only detect the set of transcripts chosen for analysis, whereas 'open' *e.g*. tag-based technologies are capable of identifying all possible transcripts, including those that were previously uncharacterized. Although new technologies are now emerging, at present the major resources for open-type analysis are the many publicly available SAGE (serial analysis of gene expression) and MPSS (massively parallel signature sequencing) libraries. These technologies have never been compared for their utility in the context of deep transcriptome mining.

**Results:**

We used a single LongSAGE library of 503,431 tags and a "classic" MPSS library of 1,744,173 tags, both prepared from the same T cell-derived RNA sample, to compare the ability of each method to probe, at considerable depth, a human cellular transcriptome. We show that even though LongSAGE is more error-prone than MPSS, our LongSAGE library nevertheless generated 6.3-fold more genome-matching (and therefore likely error-free) tags than the MPSS library. An analysis of a set of 8,132 known genes detectable by both methods, and for which there is no ambiguity about tag matching, shows that MPSS detects only half (54%) the number of transcripts identified by SAGE (3,617 versus 1,955). Analysis of two additional MPSS libraries shows that each library samples a different subset of transcripts, and that in combination the three MPSS libraries (4,274,992 tags in total) still only detect 73% of the genes identified in our test set using SAGE. The fraction of transcripts detected by MPSS is likely to be even lower for uncharacterized transcripts, which tend to be more weakly expressed. The source of the loss of complexity in MPSS libraries compared to SAGE is unclear, but its effects become more severe with each sequencing cycle (*i.e*. as MPSS tag length increases).

**Conclusion:**

We show that MPSS libraries are significantly less complex than much smaller SAGE libraries, revealing a serious bias in the generation of MPSS data unlikely to have been circumvented by later technological improvements. Our results emphasize the need for the rigorous testing of new expression profiling technologies.

## Background

In recent years, a number of techniques have emerged for large-scale gene expression analysis. Most are designed to compare the expression of many genes between cell types or under a number of different conditions. However, there has also been interest in techniques capable of identifying the complete transcriptome of a given cell or tissue. 'Closed' architecture systems, such as microarrays, are less suited to this application because they are limited by the extent to which global transcriptome coverage has been achieved. Even in organisms such as *Homo sapiens *where a complete genome sequence is now available, there remains uncertainty regarding the actual number of transcribed regions. This is true in the case of conventional genes and even more so if regions thought to yield polyadenylated non-coding RNAs are included [1-3]. Thus, at the present time, it would in principle be necessary to represent the whole genome on an array in order to test for all possible transcripts, which presents two major difficulties. First, there is the shear number of probes required to fully cover the human genome using tiling arrays: 51,874,388 probes on 134 arrays were required even for non-overlapping coverage of non-repetitive regions in a study undertaken in 2004 [2]. Second, there are the technical difficulties associated with designing consistently good probes covering the whole genome (discussed in, *e.g*., [4]). It may, therefore, be some time before all human genes can be confidently sampled in a conventional laboratory setting using such methodologies.

Much use has therefore been made of 'open' gene-expression profiling methods requiring no *a priori *knowledge of the genes likely to be of interest [5]. Many of these techniques are based on the sequencing of short tags created from pooled transcripts. Until recently, tag-based expression profiling technologies had a key advantage over more traditional 'open' technologies such as expressed sequence tag (EST) or cDNA sequencing insofar as they efficiently and relatively inexpensively sample large numbers of transcripts. In SAGE, between 12 and 20 transcripts are sampled per sequencing reaction, compared to one EST or a fraction of a cDNA, whilst in MPSS all tags in a library (usually >1 million) are sequenced simultaneously. New sequencing techniques, such as LCM-454 technology [6], may allow rapid sequencing of very large EST libraries [7], but these may lack the quantitative nature of tag-based techniques because production and capture of the ESTs are likely to be length and/or sequence dependent. These technologies could, however, be used to sequence extremely large SAGE libraries.

An additional advantage of 'open' technologies is that sensitivity can be improved to a great extent simply by increasing library size, allowing the identification of very weakly expressed transcripts. Such transcripts may be expressed at levels much less than one copy per cell because they are only present at, *e.g*., very specific points in the cell cycle or in response to particular levels of cellular stress that only apply to subsets of the cell population. One caveat to this is that background noise in the data, *e.g*. due to contaminating species, degradation or mis-priming, may limit the maximum sensitivity that can be achieved (for an example of how this can affect comparative analyses, see [8]). In contrast, for microarrays, sensitivity is limited by the inherent signal:noise ratio of the read-out technology itself, rather than only biological noise. Best estimates of the sensitivity for cDNA or long oligonucleotide arrays vary from 50 to 400 transcripts per million, whereas, using the same type of analysis, species present at an average count as low as 5 transcripts per million could be reliably identified as being differentially expressed in large-scale tag-sequencing experiments [9]. Estimates of detection sensitivity for short oligonucleotide arrays have not been calculated in the same manner, but others have claimed the reliable detection of transcripts expressed as weakly as 6–20 per million using Affymetrix GeneChips [10]. However, this sensitivity was somewhat dependent on comparisons to a 'mismatched control' oligonucleotide, the results of which were compromised by variable cross-reactivity with the mismatched oligonucleotide.

The disadvantage of sequencing very short tags is that it compromises identification of the transcripts corresponding to each tag. Ideally, every tag would map uniquely to both the genome and the transcriptome, and every transcript would be represented by at least one tag. Short sequence tag-based profiling was pioneered by Velculescu *et al*. in the form of conventional SAGE [11], which produces 14 bp tags from the 3'-most occurrence of an "anchoring" restriction site (usually *Nla*III) in polyadenylated transcripts. This might be thought to be sufficient to map uniquely to the transcriptome [11], but because transcript sequences are non-random, such tags are often too short to distinguish similar sequences. In addition, genomic mapping of the tags usually generates multiple hits, making the identification of novel genes extremely difficult.

Other tag-based methodologies, especially those for gene identification and establishing transcriptional start points, have since been developed that generate longer tags from the 3' or 5' ends of transcripts, or both (reviewed in [12]). Until now, the most common techniques that have been used for tag-based global expression analysis are LongSAGE [13] and massively parallel signature sequencing (MPSS) [14]. Little, if any, data has been generated or made available in the public databases with the newer sequencing technologies, such as LCM-454, which would in principle allow rapid production of extremely large EST and SAGE libraries [7] or Solexa's SBS technology [15], which has been adapted to tag-based expression profiling [16].

LongSAGE is a modification of the standard SAGE protocol using a different type II restriction enzyme (*Mme*I rather than *BsmF*I) to generate a 21 bp tag at the anchor site (which remains *Nla*III). MPSS generated 20 bp tags anchored at the 3'-most *Dpn*II sites in transcripts, in a similar manner to SAGE. The unique feature of MPSS was the proprietary, bead-based sequencing technology, which was more efficient than standard Sanger sequencing and yielded far larger tag counts. As both methods significantly increase tag length compared to conventional SAGE, they were expected to improve the prospects for unique genome and transcriptome tag mapping, as suggested by the pilot-scale use of LongSAGE for genome annotation [13]. As a proprietary technology that the parent company has ceased to offer, new MPSS libraries can no longer be generated. Nevertheless, large amounts of MPSS data are still being made available (see *e.g*. [17]).

Although the ability of the MPSS and LongSAGE methods to identify abundant or differentially expressed genes has been compared, their capacity to provide complete transcriptome coverage has not. The number of transcripts expressed in a single cell can vary considerably depending on cell type, among other factors, but it has been estimated that a 'typical' human somatic cell contains ~400,000 mRNA molecules [18]. Given that a particular transcript species could be present at less than one copy per cell, *i.e*. less than two tags per million (tpm), full transcriptome coverage using tag-based methods can only be guaranteed if libraries containing several times this many tags are fully sequenced. Due to the efficiency of MPSS sequencing, it became feasible to sequence well in excess of 1 million tags per sample at a fraction of the cost of sequencing a similar number of LongSAGE tags. It has seemed, therefore, that MPSS was the technology most likely to offer the depth of sampling required for whole transcriptome coverage, but this has not been adequately tested.

We previously analysed a CD8^+ ^T-cell clone using conventional SAGE [19] and found that a library of only 71,174 tags contained sequences corresponding to most, if not all, the transcripts encoding the surface molecules from that cell. However, some of these tags were found only once in the library and it is likely that transcripts from many other functional classes were not sampled at all. Similar-sized libraries have been generated from other leukocyte populations [20-22], and extensive microarray analysis has identified large numbers of transcripts differentially expressed among leukocyte subsets [23,24]. Herein, we compare the ability of SAGE and MPSS data to provide, as far as is currently feasible, access to the entire transcriptome of a T cell.

## Results

### Systematic limitations

Before undertaking a direct comparison of the two transcriptome-profiling methods, we consider the systematic limitations of the methods, as previously done in a generalised way [25]. First, for a restriction site-based tagging method to detect a given transcript, the transcript must contain that site. Using *Nla*III (as in SAGE) or *Dpn*II (as in MPSS), which each have four-base recognition sites, the recognition site ought to be present, on average, every 256 base pairs. However, some transcripts will not have these sites and both SAGE and MPSS are expected to be similarly affected. There are 13,665,294 and 410,369 *Nla*III sites in the human genome and in all the human sequences in Release 19 of the RefSeq database [26], respectively. The numbers for *Dpn*II sites are 7,112,355 and 253,936, so this site is rarer, suggesting that the ability of MPSS to tag more transcripts is in this way compromised. In RefSeq, excluding predicted transcripts from the genome, the proportion of cDNAs lacking the LongSAGE recognition site is less than 0.6% (144/24,261) whereas the proportion lacking the MPSS site is substantially higher, at ~2.3% (552). In terms of the total pool of transcripts, these numbers are relatively small, but cannot be overlooked if the entire transcriptome of a cell is to be identified. A better strategy would involve a combination of sites: only 39 of the 24,261 human sequences in RefSeq Release 19 lack both *Nla*III and *Dpn*II recognition sites.

A second limitation of tag-based methods is the difficulty of matching each tag to a unique transcript. The single most important benefit of open expression technologies is their ability to identify previously uncharacterised genes, which requires that novel tags can be linked to sequenced transcripts or, if they have not been previously identified, to the genome. Analysis of *Nla*III and *Dpn*II sites in the human genome demonstrates the effect of tag length on transcript identification [see Additional file 1]. The vast majority (>95%) of all potential LongSAGE and MPSS tags are unique in the genome and transcriptome, compared with only 9% of potential conventional (14 bp) SAGE tags, indicating that these technologies significantly reduce the problem of unique transcript identification. These results reinforce and extend the results of Unneberg *et al*. [25], obtained before LongSAGE was in use, which suggested that tags of at least 17 bp would be needed to find unique matches among human Unigene clusters. Nevertheless, the identification of novel genes using LongSAGE and MPSS tags is not straightforward, because apparently novel tags may arise via sequencing errors or genetic polymorphisms. The combination of LongSAGE and MPSS data should provide a powerful approach for identifying new transcriptional loci, since regions of the genome that are not known to code for any genes, but which contain matches to tags derived by both methods, would be highly likely to encode novel transcripts.

### Library production

LongSAGE and MPSS libraries were prepared from a single sample of RNA extracted from a CD4^+ ^T-cell clone (clone 29) activated with beads coated in anti-CD3 and anti-CD28 antibodies. Clone 29 was derived from the peripheral blood mononuclear cells of a subject given a modified vaccinia virus Ankara (MVA) vaccine containing a polyprotein made from HIV-1 gag fused to a string of cytotoxic T-cell epitopes as part of a vaccine trial [27]. It was established in culture with IL-7 and the overlapping HIV-1 gag peptides, KRWIILGLNKIVRMY and GEIYKRWIILGLNKI, but was shown to respond specifically to the former peptide. The likely clonality of the population was confirmed by analysis of TCR Vβ chain usage, which showed exclusive expression of Vβ17 (JS, SRJ and SHIA, unpublished). A single library of 503,431 LongSAGE tags, and 3 libraries containing a total of 4,274,992 MPSS tags, were sequenced.

FACS analysis indicated that, prior to library generation, the activated T-cell clone expressed CD4, CD28, CD45 and CD69, but not CD27 or CD62L (data not shown). The LongSAGE data perfectly matched the FACS results and revealed the expression of each of the classical T-cell markers, *i.e*. all TCR/CD3 components, CD2, CD4, CD5, CD6, CD11a (LFA-1a), CD43, CD45 and CD53. The MPSS library, however, lacked tags corresponding to both CD3γ and CD69. The LongSAGE CD69 transcript tag derived from the 3' untranslated region (UTR), upstream of the only *Dpn*II site in the full length cDNA. Between the *Nla*III site and the *Dpn*II site there is a potential polyadenylation signal, suggesting that alternative polyadenylation could be responsible for the absence of a CD69 MPSS tag. Even though CD3γ is the most weakly expressed transcript of those tested here, the lack of any MPSS tags derived from transcripts of CD3γ is very surprising, given the supposedly increased depth of the MPSS libraries compared to SAGE. Taken in isolation, this finding could have implied that there is an additional region of the CD3γ 3' UTR containing a potential MPSS tag that is not recorded in the main DNA sequence databases. However, in a second MPSS library made from the same mRNA sample the CD3γ tag was represented at 9.5 tags per million. Thus, the tag is produced and, given its expression level, should be found in every library of this size provided that every transcript is equally likely to be sampled. This provided the first indication of MPSS sampling problems, despite the size of MPSS libraries.

### Analysis of known genes

Ideally, the level of expression of every distinct transcript identified by the two methods would be compared. However, ambiguities in tag to gene mapping and differences in tag anchoring sites mean that different populations of potential tags will be sampled in each case, making such comparisons non-trivial. Therefore, a set of test transcripts that contain both *Nla*III and *Dpn*II sites, and for which the potential tags at all such sites are unique in both the human genome and the Ensembl transcriptome, was extracted from Ensembl [28]. This set was called UTBS (Unique Transcripts for Both Sites) and consisted of 8,132 transcripts. The Spearman correlation coefficient for expression of UTBS transcripts in the two libraries was 0.66. As expected, this correlation is significantly higher than that obtained for comparisons of the MPSS library with other, *i.e*. non-CD4^+ ^T cell-derived LongSAGE libraries; for example comparison with an activated CD8^+ ^T cell-derived LongSAGE library (83,553 tags; SHIA *et al*., unpublished) yielded a correlation of 0.55. Importantly, however, the correlation between libraries produced from the one RNA sample using the two methods was far lower than that for LongSAGE libraries produced from distinct cell populations. The coefficient obtained for a comparison of our activated CD4^+ ^T-cell LongSAGE library with the activated CD8^+ ^T-cell library referred to above, for example, is 0.76 and when our library is compared to a second LongSAGE library of similar size generated from the same cells in the "resting" state, *i.e*. prior to activation with anti-CD3 and anti-CD28 antibody coated beads (501,343 tags; MTV *et al*., unpublished), the correlation coefficient is 0.88.

Given that both methods are believed to be generally reproducible [29-31], the larger-than-expected differences between the LongSAGE and MPSS libraries generated from the same RNA sample is suggestive of a systematic bias intrinsic to one or other of the methods. To identify the source of this bias, and to establish which of the methods is the more reliable, the libraries were compared at the levels of sampling depth and breadth.

### Depth of sampling

**Figure 1:**
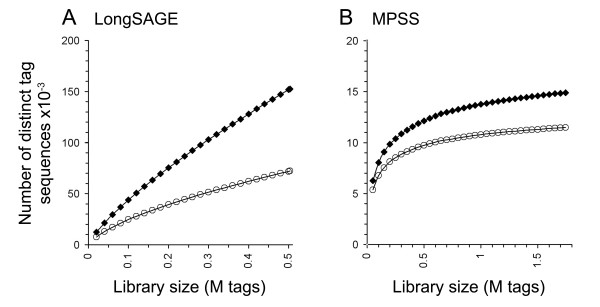
*Effect of total number of tags sequenced on number of distinct tag sequences identified*. LongSAGE (***A***) and MPSS (***B***) libraries produced from an activated CD4^+ ^T-cell clone were sampled at various sizes to examine the effect of library size on the number of distinct tag sequences identified. If the library is large enough to sample all available tags, then increasing the library size will not increase the number of sequences detected. Closed diamonds represent all tags in the library. Open circles represent only those tags that exactly match either the genome or the transcriptome (*i.e*. excluding possible sequencing errors but also polymorphisms and some tags crossing splice junctions).

**Figure 2:**
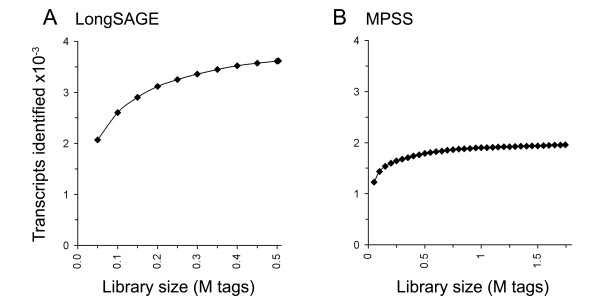
*Number of transcripts in the UTBS dataset identified by LongSAGE and MPSS*. The UTBS dataset consists of transcripts containing both *Nla*III and *Dpn*II restriction sites and for which all extracted tags are unique in both the transcriptome and the genome. The LongSAGE (***A***) and MPSS (***B***) libraries were sampled at various sizes and the numbers of transcripts from the UTBS dataset for which tags were identified were calculated.

The rate of addition of novel tag sequences to the library provides a measure of whether a given library is large enough to identify every potential tag sequence in the initial sample, since, when all existing tags have been sequenced, this rate should approach zero. As expected, given their relative sizes, this appears to be the case for the MPSS but not the LongSAGE library. However, the rate of novel tag addition is likely to be artificially increased in the LongSAGE library due to sequencing error accumulation [32]; MPSS has been reported to have much lower error rates than LongSAGE, *i.e*. ~0.25% [33] versus ~0.7% per base [34]. A simple filter was used to remove tag sequences generated by errors: *i.e*. only tags that matched either the genome or the known transcriptome were kept. Some genuine tags carrying polymorphisms unrepresented in the databases, or for which no cDNA sequence is available and a splice junction or polyadenylation occurs within the tag, are likely to be removed. However, as these are comparatively rare events, this is not expected to have a large effect on library complexity [35]. Removal of the error-derived tags dramatically reduces the rate of novel tag addition in the LongSAGE library (Fig. 1), although it is still not asymptotic. The abundances of tags corresponding to UTBS transcripts were used to examine the effect of library size on the sampling of known genes (Fig. 2). It is clear from this that the MPSS library has sampled virtually all UTBS transcripts present, and that the rate of transcript discovery by LongSAGE falls to a very low level, suggesting that the LongSAGE library is probably large enough to identify most known transcripts within the transcriptome of this cell.

The analysis using all tags matching the genome (Fig. 1) versus that based on the UTBS transcript set (Fig. 2) provide different answers to the question of how many tags need to be sequenced in order to sample the entire transcriptome. Both methods suggest that the MPSS library is large enough to sample all the readable sequence species present on the microbeads (*i.e*. the number of unique sequences identified has reached its maximum). On the other hand, while the all-tag analysis suggests that a LongSAGE library needs to be substantially larger than 500,000 tags to sample all transcripts in the cDNA pool, the analysis of known genes does not. This difference is not surprising because known genes are likely, on average, to be expressed at a higher level than novel transcripts, aiding their initial identification [36,37]. However, it is also possible that many LongSAGE tags are derived from unconventional, *i.e*. non-protein encoding, transcriptional units absent from gene databases. In this case, larger SAGE libraries would be required to identify a full set of such unconventional transcripts.

### Breadth of sampling

Great sampling depth is only of value if the open expression technology identifies transcripts irrespective of their sequence. There is a large discrepancy in the number of different sequences identified by the two methods. At the same sampling depth (*i.e*. 500,000 tags) there are many more distinct tag sequences in the LongSAGE library than in the MPSS library (151,794 vs. 12,140). Allowing for differences in sequencing error rate by considering only tags that match the human genome, LongSAGE identifies 7.4-fold more unique tag sequences than MPSS (71,838 vs. 9,723). Even using the entire MPSS library, which is 3 times the size of the SAGE library, MPSS identifies 6.3-fold fewer tags than SAGE. Although up to half of this difference may be accounted for by the lower number of *Dpn*II sites in the genome, a ~3-fold reduction in number of distinct species identified by a method intended to analyse samples to a greater depth is unexpected. This large difference suggests either that the LongSAGE library contains many spurious tags randomly matching genomic sequences or that the MPSS library lacks many genuine tags, despite the sequencing of tags from every captured transcript.

A simple explanation for the greater complexity of the LongSAGE library is that SAGE samples more tags per transcript than MPSS. It is not possible to directly convert the number of tag sequences found in a library to the number of genes being profiled, for two reasons. First, most genes will have multiple tags owing to polymorphisms, alternative polyadenylation, internal polyadenosine stretches, antisense expression and incomplete cleavage by the restriction enzymes. Second, in some cases, especially within gene families, multiple genes will share the same tag sequence. It should be possible, however, to make a rough estimate of the number of transcriptional loci identified by examining the number of 'tag clusters' found in the genome. As we wanted to compare the two methods rather than identify specific genes or determine an exact gene number, a very simple set of criteria was used to define a tag cluster. Briefly, all the tags matching the genome only once were sorted by chromosome position. Each tag match was analysed in turn and was considered to be part of a new transcriptional locus if it was greater than X bases from the nearest previous tag match on the chromosome or more than Y bases from the first tag match in the previous transcriptional locus. Various values were used for X and Y, but regardless of the exact value used, the LongSAGE library identified 2.8–3.8 fold more clusters, and therefore presumably loci, than the MPSS library [see Additional file 2]. For example, if X is 10 kb, LongSAGE identifies ~27,000 loci and MPSS identifies ~8,200 loci regardless of the value of Y, once the data have been corrected for tags that had to be excluded from the analysis because they matched multiple loci. Thus, the difference in the number of tag species identified by the two methods probably does reflect a real difference in the number of expressed genes sampled, rather than a trivial difference in the number of potential tags sampled per transcript.

Examination of a set of known transcripts should help determine whether these differences are due to erroneous LongSAGE tags or to the absence of genuine tags from the MPSS library. Analysis of the tags matching the UTBS transcript set yielded the same trend as the analysis of all tags, with the LongSAGE library identifying almost twice as many UTBS transcripts as the MPSS library (Fig. 2). This data can be extrapolated to estimate the number of genes sampled in each library: since 17.3% of all the tag sequences in the MPSS library represented 1,955 known transcripts and 7.0% of the LongSAGE tag sequences represented 3,617 transcripts, it can be estimated that ~11,300 transcripts are sampled in the complete MPSS library and ~51,700 in the entire LongSAGE library. These numbers are expected to be underestimates given that known transcripts are likely to be expressed at a higher level than uncharacterized transcripts. However, these numbers are much higher than those obtained when estimating the numbers of transcriptional loci even using a maximum distance between tags (X) of just 5 kb [see Additional file 2]. The likely explanation for this is that sequencing errors are artificially increasing the number of apparently unique tags in the libraries. Using matches to the genome to define genuine tags, extrapolation from the number of UTBS transcripts found suggests that SAGE identified a total of ~24,600 transcripts and MPSS ~8,700 transcripts, in good agreement with the estimates of loci number allowing 5,000–15,000 bases between tags defining each locus [see Additional file 2].

However, the data is analyzed, MPSS seems to underestimate transcriptome complexity. In this context, it is revealing to examine the expression level of the different classes of transcripts in the UTBS dataset. The average representation level for all LongSAGE tags corresponding to each sense transcript in this set (3,617 transcripts expressed in total) is 45 tags per million (tpm), whereas for transcripts identified by both methods the average total SAGE tag count for a transcript is 65 tpm (1,855 transcripts) and for those identified by LongSAGE only, it is 23 tpm (1,762 transcripts). This suggests that MPSS fails to detect weakly expressed transcripts. Since this is not what is expected of a method capable of sampling many more tags than SAGE, it implies that there are systematic biases in MPSS sequencing, or in library production, or both.

A trivial explanation for these results is that there is DNA contamination of the LongSAGE library but not the MPSS library. It is of course very difficult to prove that there has been no contamination of a library when deep transcriptome analysis of the given cell has not been undertaken previously. Clearly, every care was taken to ensure that there was no contamination of the libraries at any stage. However, if the SAGE library was contaminated after the initial RNA sample was divided, there are three possible sources of contaminating DNA that could explain our results (*i.e*. that generated matches to the human genome): human genomic DNA, DNA from other human transcripts or ditags from previously generated human SAGE libraries. The only LongSAGE library previously produced in our laboratory was derived from anti-CD3 antibody-treated CD8^+ ^T-cells (SHIA *et al*., unpublished). In this library, there are very high tag counts for tags derived from transcripts encoding CD8 (934 tpm total) and several other molecules that are completely absent from the CD4^+ ^T cell-derived SAGE library. Similarly, tags that are extremely abundant in both the activated CD8^+ ^T cell-derived library and our activated CD4^+ ^T cell-derived library are completely absent in another large resting CD4^+ ^T cell-derived library (MTV *et al*., unpublished), *e.g*. CCL4L1 at 1688 tpm, 2029 tpm and 0 tpm, respectively. Thus, library cross-contamination seems unlikely. In addition, the new libraries did not contain any tags derived from transcripts encoding markers of cells that are likely sources of cDNA contamination in our laboratory, *e.g*. B cells (CD19, CD20, CD21, CD22), myeloid cells (CD14, CD32) or keratinocytes (KRT5, KRT9, KRT14, KRT17). Finally, in the case of genomic DNA contamination, the abundance of contaminating tags would be expected to correlate directly with the number of copies of that sequence found in the human genome. However, the abundance distributions for SAGE tags from UTBS transcripts detected by SAGE only is equivalent to that of all the tags matching UTBS transcripts detected by SAGE as well as those detected by SAGE and by MPSS [see Additional file 3]. Thus, there do not appear to be any differences in the distribution of tags detected only by SAGE that can be attributed to genomic contamination.

### Analysis of MPSS bias

**Figure 3:**
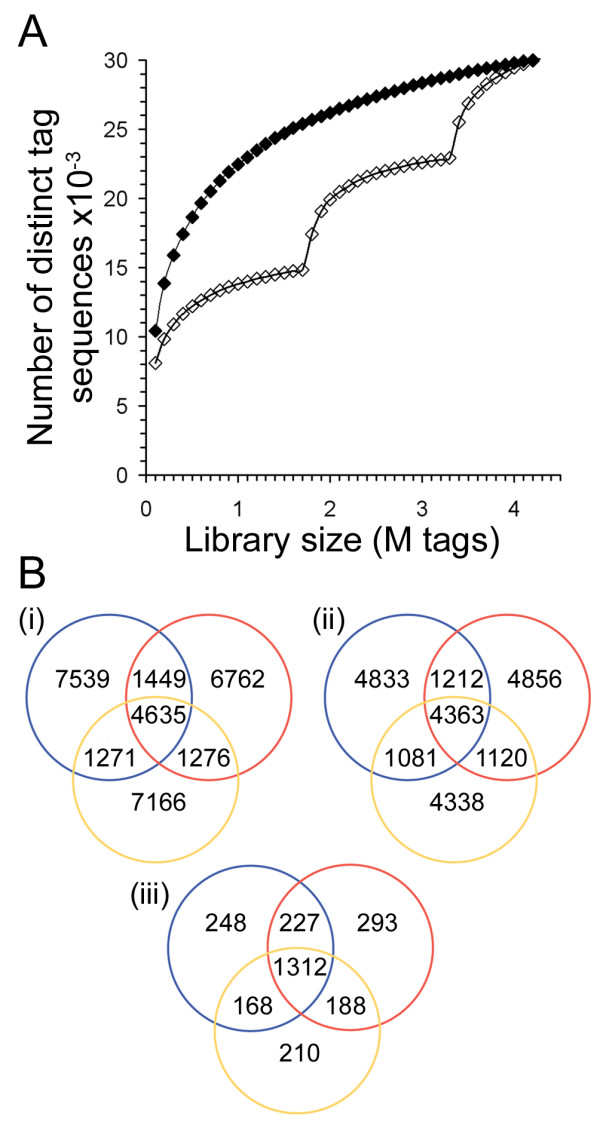
*Comparison of tag sequences in three MPSS libraries produced from the same RNA sample*. **A. **The three libraries were sampled to various sizes in a step-wise fashion to examine the effect of library size on the number of distinct tag sequences identified (as done for single SAGE and MPSS libraries in Fig. 1). Closed diamonds represent random sampling of tags from all three libraries combined. Open diamonds represent sampling of each library in turn. Clearly, although the number of distinct species identified by each library (with the possible exception of the third) appears to approach saturation, each library is sampling a different subset of sequences from the initial RNA pool. **B. **Venn diagrams showing the distribution of tag sequences between the three MPSS libraries. The library represented by the blue circle is the one used in most of the analyses presented in this study. Diagram (i) represents all the different tag sequences in the libraries. Diagram (ii) represents only those tags that match the genome; this reduces the influence of sequencing errors. In both comparisons, the majority of distinct sequences are found in only one library. Diagram (iii) represents known transcripts in the UTBS dataset found expressed in the sense direction. Here the pattern is less marked, but still only half the transcripts were observed in all three libraries (1,312/2,646). The improvement in the correlation of the libraries for known transcripts (*i.e*. those in the UTBS) was expected because more highly expressed transcripts are more likely to have been previously identified, and therefore known transcripts tend to be more abundant and have a greater chance of being sampled.

In order to try to identify the nature of MPSS bias, we analysed additional MPSS libraries and examined the complexities of one library at different stages of sequencing. Two more MPSS 'bead libraries' were produced from the same cDNA sample used in the production of the library considered up to this point. Since analysis of the first bead library revealed substantial sequence redundancy, *i.e*. virtually no new sequences were added as the library size increased (Fig. 1), we expected the tag composition of additional bead libraries to be essentially identical. Instead, addition of tags from the new libraries causes dramatic increases in the number of different species (Fig. 3A). Each library has similar numbers of distinct tag sequences (~14,100 to ~14,900 per library), but the majority (71%) of these are only found in one of the three libraries, even after excluding tags that do not match the genome (*i.e*. potential sequencing errors; Fig. 3B). The tags found in any one library are present at much lower levels than those found in all three libraries (*i.e*. averaging 9.4 tpm vs. 191.3 tpm). This suggests that random sampling during MPSS library preparation has a large effect on the resulting 'bead library', profoundly reducing its complexity. Since only 2,646 transcripts are identified in the UTBS dataset when the three MPSS libraries are combined (Fig. 3B), versus the 3,617 identified by LongSAGE, more than three MPSS libraries would be required for comprehensive transcriptome analysis using MPSS.

The raw MPSS data was provided in three forms, extracted at different stages of sequencing: *i.e*. after the sequencing of tags of 14 bp, 17 bp and 20 bp. A comparison of the alternate tag extractions from the first bead library (1.74 M tags) suggests that sequencing length has an effect on the complexity of the library: the longer the tag sequence, the smaller the number of unique tags that are sequenced (Table 1). The 14 bp library was ~24% more complex than the 20 bp library, contrary to expectation: a 14 bp library generated *in silico *from the 20 bp data is ~16% less complex than the 20 bp tag library. It is possible that the additional 14 bp tag sequences that are absent from the libraries of longer tags are 'bad sequencing reads' that are filtered out in the last rounds of sequencing. If this were the case, the 20 bp library should constitute an improvement on the libraries of shorter tags, and a larger proportion of the long tags ought to match the genome sequence. Instead, we found the opposite: as tag length increased, a smaller proportion of tags matched the genome (Table 1). There are two explanations for the apparent drop in 'sequence quality' as tag length increases. First, as tag length increases, the chance that an error, polymorphism or splice junction may occur within the sequence also increases. Second, shorter tags are more likely to match the genome due to chance even if they contain an error (analogous to the chance of a genuine tag from one gene randomly matching other locations in the genome; see Additional file 1). The loss of library complexity during successive sequencing cycles can only exacerbate the much larger loss of complexity resulting from sampling error at the stage of bead library construction revealed by our comparison of multiple MPSS libraries.

**Table 1:** Effect of tag length on MPSS library complexity

Tag length sequenced (bp)	Length of tags analysed	Number of unique tags	Tags matching genome sequence
20	20	14,894	11,489 (77%)
20	17	13,576	11,934 (88%)
20	14	12,509	12,372 (99%)
17	17	18,084	14,307 (79%)
17	14	15,190	14,944 (98%)
14	14	19,931	19,402 (97%)

MPSS tags can be extracted from the same initial dataset to produce tags of different lengths; in this case 14, 17 and 20 bp tags were extracted. After the extractions, tag lengths can be computationally shortened to see if there is a difference in complexity between the different tag extractions. Decreasing the tag length sequenced was, unexpectedly, found to increase the complexity of the library. For example, 14,894 different 20 base tags were produced, which contained 13,576 different 17 base sequences if the last 4 bases were ignored. However, if the tags were initially extracted at 17 bases (*i.e*. ignoring the last annealing step in sequencing) then a library of 18,084 different tag sequences was produced; 4,508 distinct species are therefore lost in this last sequencing step. The last column shows how many of the distinct tag species have perfect matches in the human genome, and this is also expressed as the proportion of the species identified (in brackets).

### Identification of potential novel transcriptional loci

The deep sampling by LongSAGE and to a smaller degree by MPSS means that tags can be matched to genomic regions where transcripts have not previously been identified or predicted by Ensembl (>10,000 tags match such regions). However, many of these matches are unlikely to correspond to actual transcriptional loci, as tags may match more than one genomic site or may represent sequencing errors arising fortuitously from more abundant tags that match the genome elsewhere. On the other hand, it is likely that loci identified by both methods will represent genuine regions of transcription. To investigate the likely numbers of new transcriptional loci identifiable using this approach, strict criteria were used to identify regions where transcription was detected by both methods. Tags were required to match the genome only once, at a position where no known Ensembl genes are annotated within 5000 bases in the sense or antisense direction. Of all the tags, only 5, 975 unique LongSAGE tags and 392 MPSS tags satisfied these criteria (using only the first of the three MPSS libraries). The genomic matches to the tags in both lists were then examined in order to ascertain whether they could be part of the same gene. If a LongSAGE tag matched the genome within 5,000 bases of, and on the same strand as, an MPSS tag, this pair of tags was considered to define a potentially new transcriptional locus. This procedure identified only 147 tag pairs, none of which occur within 5,000 bases of predicted genes in Ensembl Release 40 (*i.e*. genes predicted without direct cDNA sequence data *e.g*. from comparison to other genomes). These loci therefore represent possible transcriptional loci for which no clear evidence has previously been obtained. The pairs of tags are listed in Additional file 4. Interestingly, the average abundance of MPSS tags that match the genome once and have some form of gene annotation is 59.8 tpm but for those in this novel gene list, it is 20.2 tpm. This confirms that novel genes tend to be expressed at a lower level than those already discovered. It is also interesting to note that roughly half these tag pairs (*i.e*. 72) are found in genomic regions masked in Ensembl, which are more difficult to analyse by other methods owing to the presence of repetitive elements. Identifying the transcripts corresponding to all these novel loci should be relatively simple using both tags as primers for direct PCR or nested 5' rapid amplification of cDNA ends (RACE) [38]. Overall, however, our observations suggest that relatively few *bona fide *new transcriptional loci remain to be discovered.

## Discussion

We have described the production of large LongSAGE and MPSS libraries from a single RNA sample and consider their usefulness for identifying the complete transcriptome of a clonal population of cells, including transcripts not expressed in all cells and hence present on average at less than one copy per cell. The two methods give very different estimates of the number of genes expressed by a single cell. Both by counting the number of genomic loci represented or by extrapolation from the number of known genes found, the SAGE tags sequenced are estimated to represent 20,000–30,000 transcripts, whereas the MPSS tags represent 7,000–9,000 transcripts. The total number of genes in the human genome is still being debated, but the current consensus places it under 30,000 protein encoding genes [39] (and perhaps below 25,000 [40]). Estimates of the number of different transcripts expressed in a single cell vary widely. Early studies on mouse brain suggested that there are between ~10,000 [18] and ~100,000 [41] transcript species per cell. However, gene expression is a stochastic process [42], so it might be expected that, if a pure population of cells could be sampled deeply enough, transcription from every gene would be detected.

The main conclusion of our work is that although MPSS yields large amounts of expression data very rapidly, sampling problems severely limit transcriptome coverage and bias library complexity towards genes transcribed at higher levels. Our analysis suggests that, despite the very significant problem of sequencing errors in large SAGE libraries, until large amounts of data from new techniques such as SBS [16] become available, LongSAGE will remain the best source of available data for the deep mining of cellular transcriptomes. In organisms for which the complete genome sequence is available, most sequencing errors can be removed by excluding tags that fail to match either the genome or any known transcript. This is likely to remove a few genuine tags because databases of expressed sequences are not complete and splicing, polyadenylation and sequence polymorphisms mean that expressed sequence is not always identical to genomic sequence. However, because most transcripts contain more than one SAGE tag, very few expressed genes will remain unidentified. Other methods for removing sequencing errors from SAGE libraries are mostly based on identifying tags in the library related by simple mutations (single base changes, insertions or deletions). In our hands, these methods removed several genuine transcripts of interest without removing as large a proportion of the tag sequences as the genome-based approach (data not shown). For organisms for which complete genome sequences are unavailable, only these methods would allow meaningful lists of tags representing truly novel transcripts to be compiled.

We are uncertain about the source of the reduced complexity of the MPSS data. It seems clear that a lack of sampling due to insufficient sequencing is not the major problem. For each bead library virtually all the distinct sequences are sampled after sequencing fewer tags than are obtained in an average library produced using the standard protocol. From our comparison of three MPSS libraries prepared from the same RNA sample, it would appear that the largest amount of complexity is lost at the stage of bead library preparation. Each library samples a small fraction of the transcriptome and, even in combination, the three MPSS libraries fail to identify as many known genes as an >8-fold smaller LongSAGE library, let alone as many different transcript species overall. At present it is not obvious whether it is the production of tags from cDNA or tag to bead ligation that is most responsible for reducing library complexity prior to bead loading and sequencing.

It should be noted, however, that some transcripts are lost in the course of sequencing, as demonstrated by the loss of library complexity in terms of the number of species identified as tag sequences are extended from 14 to 17 to 20 bp. We are not the first to identify such effects. MPSS sequencing proceeds in four-base steps yielding four-base "words" and it has been noted by Meyers *et al*. that MPSS has problems with palindromic words (*e.g*. TTAA) [33,43], leading to bias against detection of these sequences. However, Meyers *et al*. [33] estimated that only ~8% of sequences would be affected by this source of bias and in our libraries this effect will have been suppressed by sequencing in two staggered phases, since this increases the number of tags that do not contain 4 base palindromes in at least one of the phases. The extent of the palindrome effect is therefore not enough to explain the large decrease in library complexity we observe when comparing the 14, 17 and 20 bp extractions of the sequencing data. Either the palindrome-dependent effect is larger than expected or there is some additional, currently unidentified, systematic bias in MPSS sequencing.

A potential source of bias in classical MPSS data is that the cDNA species immobilised on the beads following *Dpn*II cleavage vary significantly in length (*i.e*. the distance between the cleavage site and the end of the cDNA). The effect of tag-position within the cDNA on the observed abundance of MPSS tags has been analysed by Chen and Rattray [44] who found that this was a significant source of bias for both "classical" and "signature" MPSS, although it was more serious for classical MPSS. Signature MPSS is a refinement of the method wherein cDNAs are cleaved with *Mme*I after cleavage with *Dpn*II and ligation of a linker so that the same length of sequence, *i.e*., the tag, is immobilised on the beads in each case. This approach is analogous to the SAGE process, which ensures that all ditags amplify uniformly. Tag-position bias is likely to affect the observed abundance of many tags in our libraries. However, if the library is sequenced to completion, as our data suggests (Fig. 1), all different tag sequences on the beads should have been sampled even if the frequency of sampling does not correlate with abundance. It is possible that there is a maximum length of cDNA species beyond which tags are never (or hardly ever) observed, but this has not been demonstrated and is unlikely to account for the level of inter-library variation we observed.

Analysis of the GC content of observed SAGE and MPSS tags, compared to that expected by random sampling of tags from known genes [45], also pointed to bias in MPSS but not SAGE tag identification. LongSAGELite [46], which has an additional amplification step, may introduce bias, however. Several signature MPSS libraries analysed by Siddiqui *et al*. [45] were found to be biased towards GC-rich tags. In contrast, the few classical MPSS libraries that were analysed seemed to have a small bias towards AT rich tags. Using a similar approach to Chen *et al*., we find that the GC content of both our SAGE and MPSS libraries is higher than that seen in random sampling by ~13 standard deviations and ~57 standard deviations, respectively. These deviations are larger than those observed by Siddiqui *et al*. [45], which may reflect differences in the details of the sampling procedure or the Refseq pool used. The difference we see between the two methods is consistent with their data for signature MPSS libraries and LongSAGE libraries, *i.e*. that the MPSS method appears to be significantly biased in favour of GC rich tags. It remains unclear whether this accounts for the complete absence of large numbers of AT rich tags from an MPSS bead library, but it at least partly explains the loss of complexity at the sequencing stage.

## Conclusion

Our results suggest that MPSS data ought to be used cautiously. Although conventionally sequenced SAGE datasets therefore constitute the only reliable sources of quantitative digital gene expression data at present, this situation is almost certainly set to change. New DNA sequencing technologies, based on polony sequencing [6,15], are likely to provide additional, very large datasets. These methods will reduce both the cost and the time taken to generate large SAGE and EST libraries, making these methods even more accessible. It is unlikely that the use of new sequencing technologies *per se *would introduce unforeseen biases into expression libraries, but our findings suggest that researchers will need to verify this. SBS has been adapted by Solexa, who also own the now redundant MPSS technology, as an alternative method for the parallel production and sequencing of signature sequences [16]. This technique is quite different from MPSS, in that it does not use beads, does not have the 4-base sequencing cycles, and will be available as an instrument rather than a "black box" service. However, when SBS signature expression libraries become available it will be necessary to exclude the unexpected, and largely unexplained, lack of complexity we have encountered in MPSS libraries.

## Methods

### Library preparation and sequencing

Our LongSAGE library was generated according to the standard protocol using the I-SAGE Long kit from Invitrogen (Groningen, the Netherlands) and was sequenced on an ABI 3700 capillary DNA sequencer (Applied Biosystems, Foster City, CA) using BigDye v3 terminators (Applied Biosystems) to a depth of 503,431 tags. MPSS libraries were produced from the same RNA sample as the LongSAGE library by Lynx Therapeutics Inc (now Solexa Inc, Hayward, CA) under their standard service agreement. They initially provided a library of 1,744,173 tags and then two further libraries of 1,573,952 and 956,867 tags, respectively; giving a total of 4,274,992 MPSS tags. All three libraries were provided as 20 bp reads (including the *Dpn*II restriction site sequence GATC) sequenced using "steppers" 2 and 4 [14,33]. On request, data from the sequencing of the initial MPSS library captured in the two sequencing cycles before the final one, *i.e*. tag lengths 14 and 17 bases including the *Dpn*II site, were also provided.

### Data storage

The data were stored as flat files or in a MySQL database as flat tables to simplify error checking and optimise the speed of access. Data were processed either using Perl and the DBI module for database interactions working with the MySQL database or using C programs working on the flat files. The programs used are currently undergoing optimisation but can be made available on request.

### Tag extractions from expression libraries

Sequencing runs from the LongSAGE libraries were initially processed with Phred to remove obvious sequencing errors [47]. When choosing a Phred setting one has to balance the need for high quality sequence against that of losing genuine sequences. Analysis of sequences of a similar length to SAGE ditags led Prosdocimi *et al*. [48] to conclude that low Phred settings allowed the optimal ratio of genuine sequences retained to errors removed. Therefore for this analysis, sequences with Phred scores of 10 and above were kept. The Phred screening and tag extraction from ditags were done using Perl scripts written by A.G. McArthur (Marine Biological Laboratory, Woods Hole, USA). Tags were imported into a MySQL database and those possibly derived from linker sequence were removed. Tag counts were then normalised to tags per million (tpm), and the un-rounded normalised counts were used for inter-library comparisons. Public LongSAGE libraries were downloaded from GEO177 and imported into the MySQL database using the same normalisation and linker removal script as for libraries sequenced in-house. MPSS tags were directly imported into the database from tag files provided by Lynx Therapeutics Inc.

### Tag extraction from the human genome and known transcriptome

The data source for genome and transcriptome data was Ensembl [49] (version 40, NCBI human genome sequence assembly 36). Tags were extracted from each chromosome using both masked and unmasked genomic sequence data and from the mitochondrial DNA at all possible restriction sites (of *Nla*III and *Dpn*II). Further information was then extracted for each tag: three windows were examined, both up- and downstream of the tag, for the presence of gene annotation (at the restriction site, up to 1,000 bases from the site and 1,001–5,000 bases from the site). For each window, all Ensembl genes and predicted genes were recorded. The genomic tags were then classed as being outside any known gene (default), or as exonic, intronic or boundary (*i.e*. crossing an exon-intron boundary), or as matching multiple genes.

Tags were also extracted from all transcripts in the Ensembl Genes dataset [50] at all restriction sites (*Nla*III and *Dpn*II) in both the sense and antisense direction. If a tag extended beyond the known 3' end of a transcript, it was extended along the genome unless the transcript was predicted to contain a polyA site (as defined in the supplementary material by Caron *et al*. [51]), in which case adenosines were added to the 3' end of the tag to complete the length of the tag.

### Tag-to-gene mapping

Information from the extractions described above was combined for automated tag-to-gene-mapping. First, frequencies for each tag, in both the genome and transcriptome, were calculated, and then each tag was matched to the genome and classified as one of the following: single match, multiple match, no match or excess matches (more than 20 hits to the genome). No further analysis was undertaken for the excess matches. For single matches and multiple matches where only one match occurred in or near a known gene, tags were further annotated as matching the gene or the region downstream of a gene in a sense or antisense direction. Tags matching the known transcriptome were also categorised as matching a known transcript in the sense or antisense direction or matching multiple known transcripts.

### Gene-to-tag mapping

The UTBS transcript set described in the text was produced by identifying all known Ensembl transcripts that contained at least one restriction site for each enzyme (*Dpn*II and *Nla*III) and for which all sense and antisense tags in all exons of the gene encoding that transcript were unique within the transcriptome and within the genome. Some tags may be absent from the genome due to splicing, polyadenylation and the fact that the genome is not complete. This set consisted of 8132 genes. For each gene, all possible tags derived using either method were extracted, and expression of the gene was calculated as the sum of the abundance of each of its corresponding tags.

### Statistical comparisons of expression libraries

Spearman correlation coefficients were calculated for the data using the statistical analysis program, R [52]. Spearman correlation coefficients are suitable for examining large-scale gene expression experiments because the calculation uses rank data rather than absolute values and is therefore not influenced by outliers [53].

## Abbreviations

SAGE, serial analysis of gene expression; MPSS, massively parallel signature sequencing; tpm, tags per million; UTR, untranslated region; UTBS, Unique Transcripts for Both Sites; RACE, rapid amplification of cDNA ends.

## Competing interests

The author(s) declares that there are no competing interests.

## Authors' contributions

LH devised the basic computational approaches, carried out the initial data analysis, identified the discrepancies discussed and drafted an initial report. VBS devised an improved bioinformatic strategy, undertook the rigorous testing of these results and participated in formulating the manuscript. MTV and SHIA designed and carried out the library production and data acquisition procedures. JKS and SLRJ produced the biological samples necessary for the work and undertook their analysis and testing. SJD conceived the study, participated in its design and co-wrote the manuscript. EJE coordinated and planned the detailed study, participated in the data analysis and interpretation and co-wrote the manuscript.

All authors read and approved the final manuscript.

## Supplementary Material

Additional file 1 Effect of tag length on frequency of matches to the genome and transcriptome. Additional figure showing a histogram of the frequencies of every tag found in the Ensembl genome and transcriptome for various combinations of tagging enzyme and tag length. Click here for file

Additional file 2 Number of transcriptional loci identified. Additional table showing the number of different active transcriptional loci identified in the same cell sample by either SAGE or MPSS according the method described in the text when various alternative parameters are used. Click here for file

Additional file 3 Comparisons of tag abundance distributions for LongSAGE tags from the activated CD4^+ ^T-cell library matching UTBS transcripts according to whether the transcripts are also detected by MPSS. Additional figure comparing the apparent frequency distributions of transcripts from known genes according to whether their corresponding tags were found by SAGE and MPSS or exclusively by one of these techniques in order to demonstrate that transcripts detected only by SAGE did not represent a fixed level of genomic contamination. Click here for file

Additional file 4 Novel loci of transcription identified by combining LongSAGE and MPSS. Additional table listing all the pairs of SAGE and MPSS tags found close together in genomic regions with no previously annotated transcriptional locus nearby. Click here for file
