# Supplementary material for: Deep analysis of cellular transcriptomes – LongSAGE versus classic MPSS
Source: BMC Genomics. 2007 Sep 24;8:333. doi: 10.1186/1471-2164-8-333 (PMC2104538; doi:10.1186/1471-2164-8-333)
Supplement: Additional file 1 — Effect of tag length on frequency of matches to the genome and transcriptome. Additional figure showing a histogram of the frequencies of every tag found in the Ensembl genome and transcriptome for various combinations of tagging enzyme and tag length. [file 1471-2164-8-333-S1.pdf]

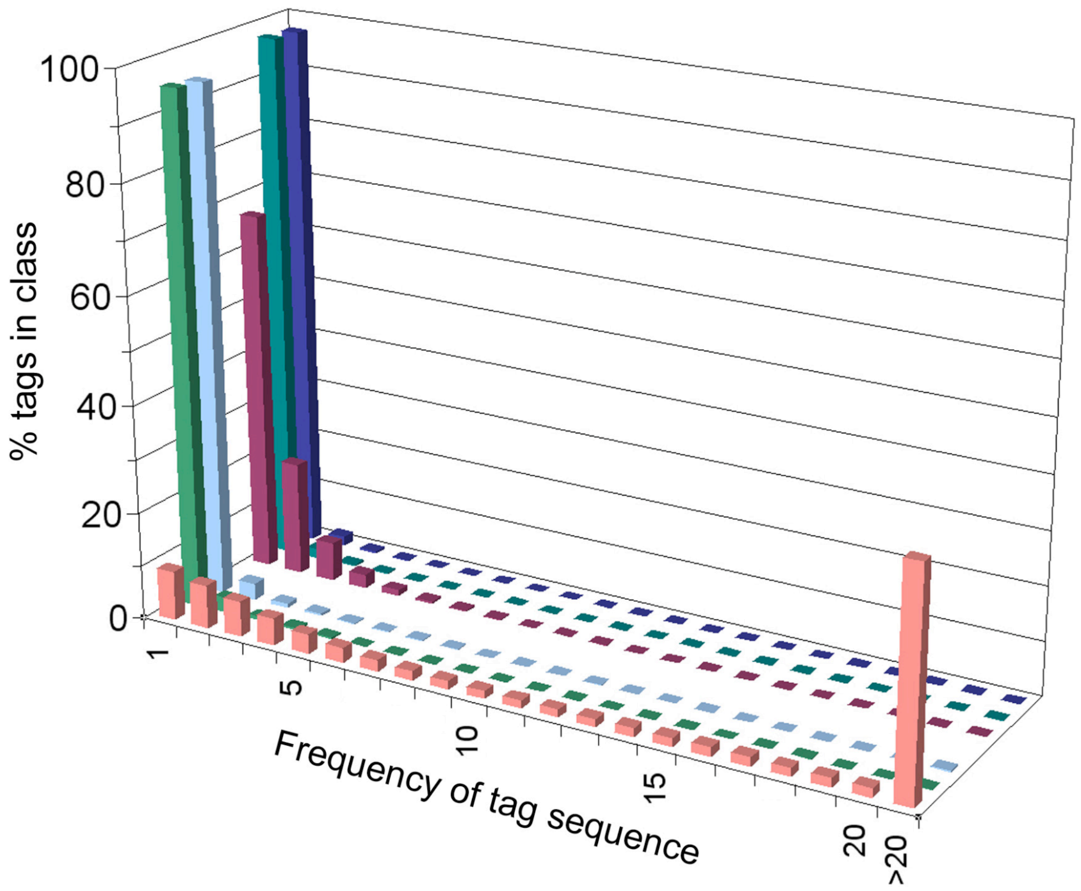

Figure S1. *Effect of tag length on frequency of matches to the genome and transcriptome.* All possible tags were extracted from the Ensembl genome and transcriptome. The frequency of each tag in the genome or transcriptome was calculated and tags were grouped by frequency. The different tag lengths chosen correspond to the different techniques used in this thesis: 14 bases, SAGE; 21 bases, LongSAGE; and 20 bases, MPSS.

	Tags Extracted From	Restriction Site	Tag Length (bases)
	Genome	CATG	14
	Genome	CATG	21
	Genome	GATC	20
	Transcriptome	CATG	14
	Transcriptome	CATG	21
	Transcriptome	GATC	20
