# Supplementary material for: Deep analysis of cellular transcriptomes – LongSAGE versus classic MPSS
Source: BMC Genomics. 2007 Sep 24;8:333. doi: 10.1186/1471-2164-8-333 (PMC2104538; doi:10.1186/1471-2164-8-333)
Supplement: Additional file 2 — Number of transcriptional loci identified. Additional table showing the number of different active transcriptional loci identified in the same cell sample by either SAGE or MPSS according the method described in the text when various alternative parameters are used. [file 1471-2164-8-333-S2.pdf]

Max. loci length (bp)	Max. distance between tags (bp)	Number of loci		Corrected number of loci		Ratio of loci found by LongSAGE:MPSS
		Long SAGE	MPSS	Long SAGE	MPSS	
50000	5000	26266	7042	33319	8974	3.71:1
50000	10000	21248	6429	26954	8192	3.29:1
50000	15000	18263	5993	23167	7637	3.03:1
50000	20000	16257	5683	20622	7242	2.85:1
100000	5000	26266	7042	33319	8974	3.71:1
100000	10000	21248	6429	26954	8192	3.29:1
100000	15000	18263	5993	23167	7637	3.03:1
100000	20000	16257	5683	20622	7242	2.85:1
150000	5000	26266	7042	33319	8974	3.71:1
150000	10000	21248	6429	26954	8192	3.29:1
150000	15000	18263	5993	23167	7637	3.03:1
150000	20000	16257	5683	20622	7242	2.85:1
200000	5000	26266	7042	33319	8974	3.71:1
200000	10000	21248	6429	26954	8192	3.29:1
200000	15000	18263	5993	23167	7637	3.03:1
200000	20000	16257	5683	20622	7242	2.85:1

Table S1: *Number of transcriptional loci identified.*

Using the location of tags that mapped *uniquely* to the genome it is possible to estimate the number of transcriptional loci. Two factors were altered to see if they had any effect on the ratio of loci found in LongSAGE vs. MPSS. The factors were: maximum loci length (variable Y in the text) and the maximum distance between any two consecutive tags in the loci (variable X in the text). The “corrected” figures take into account that in each library only a proportion of tags match the genome uniquely. The correction factors required for this step were calculated by dividing the number of unique tag sequences in the library that match the genome at all by the number that could be used in this analysis (i.e. those that match the genome *at a single unique location)*. This will be a slight underestimate, as although the majority of tag matching the genome in multiple locations will be derived from a gene at only one of the possible locations, some may be pools derived from multiple genes. The correction factors used were 1.26 for LongSAGE and 1.25 for MPSS.
