# Supplementary material for: Deep analysis of cellular transcriptomes – LongSAGE versus classic MPSS
Source: BMC Genomics. 2007 Sep 24;8:333. doi: 10.1186/1471-2164-8-333 (PMC2104538; doi:10.1186/1471-2164-8-333)
Supplement: Additional file 3 — Comparisons of tag abundance distributions for LongSAGE tags from the activated CD4+ T-cell library matching UTBS transcripts according to whether the transcripts are also detected by MPSS. Additional figure comparing the apparent frequency distributions of transcripts from known genes according to whether their corresponding tags were found by SAGE and MPSS or exclusively by one of these techniques in order to demonstrate that transcripts detected only by SAGE did not represent a fixed level of genomic contamination. [file 1471-2164-8-333-S3.pdf]

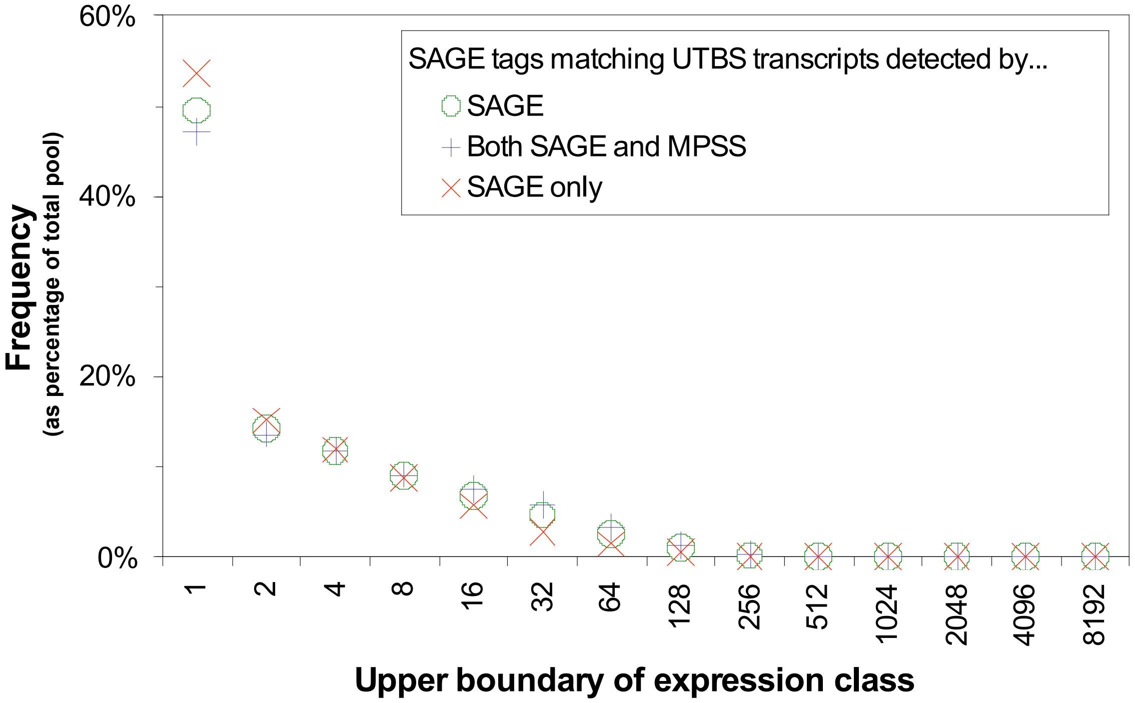

Figure S2. *Comparisons of tag abundance distributions for LongSAGE tags from the activated CD4+ T-cell library matching UTBS transcripts according to whether the transcripts are also detected by MPSS.*

Three groups of LongSAGE tags were analysed: the complete set of tags matching UTBS transcripts, the subset matching UTBS transcripts detected by both SAGE and MPSS and the subset matching UTBS transcripts NOT detected by MPSS. The abundances of LongSAGE tags were grouped into expression classes on a log base 2 scale and the percentage of the total in each class is plotted. Tags derived from transcripts detected only by SAGE appear slightly less well expressed in general, but with a similar distribution (variation in expression level) to the other groups.
