# Supplementary material for: Deep analysis of cellular transcriptomes – LongSAGE versus classic MPSS
Source: BMC Genomics. 2007 Sep 24;8:333. doi: 10.1186/1471-2164-8-333 (PMC2104538; doi:10.1186/1471-2164-8-333)
Supplement: Additional file 4 — Novel loci of transcription identified by combining LongSAGE and MPSS. Additional table listing all the pairs of SAGE and MPSS tags found close together in genomic regions with no previously annotated transcriptional locus nearby. [file 1471-2164-8-333-S4.pdf]

Chromosome	Strand	SAGE		MPSS		Distance between tags	Masked region?
		Tag sequence	Start position	Tag sequence	Start position		
1	+	GGGCCGCGAGGTTTGCA	987713	TCGGACGTGGGCTCCG	987564	149	
1	+	AAGGCACCGGCAGTCTC	7895855	CCTCTGGCTCTGCCTG	7895341	514	✓
1	+	TTTGAAAAATGAAAAAT	36308930	ATTCTTGTGGACTAGT	36309095	165	
1	+	AAGGTTGGGTAACTCCA	67530989	AGTCTTGGACCTGTGC	67534307	3318	✓
1	+	ACTTGACCAATGTCACA	143826800	TTGTCTATTGGCCCCT	143827807	1007	✓
1	+	GCTCTCTGGGACCATCA	191699505	AGTCAGGTTGACAGCT	191698809	696	
1	+	CACACGAGGGCCTTCCT	232975018	CGGCTTCCTTGATGTC	232976060	1042	
1	-	TAATAAAAGACTCCTGA	33164591	CTCCCCCACAGCCCGC	33167322	2731	✓
1	-	TTCTGGGTTATGTGCAA	111003271	CATCTGCCAGCACATA	111003616	345	
1	-	TGTCTTGTTTGGTTGCT	117138682	CTGCCCTGTAAGGAAG	117140293	1611	✓
1	-	TCAGTTTTGTGAAATAG	118207503	TGACTCCAAAGCGCAT	118207889	386	✓
1	-	ACTGAGTCGCTGGACCA	217675250	TTTTGCTGTTGTGAAG	217678695	3445	✓
2	+	CAGCGCTGCCGTGGACG	7788191	TGTCTCTCTTGCATAT	7788211	20	✓
2	+	CATAAATAAACCTTTAT	7818403	CCTAGTCCACAGAAAT	7817933	470	✓
2	+	TTGCTTCTTCAACAAAT	61011554	CCAGCAGAATACCAAA	61011983	429	
2	+	GACAGAATTTTTTTTGT	122239596	TCTCCATCCGGGGCTT	122239357	239	
2	+	TAAATACTGTTGGTGGT	122240620	ATCCCACAGACAATAC	122241086	466	
2	+	AATTTTTTTGGCCTTTT	179088953	ACCCTTAGTCATCACT	179087739	1214	
2	+	GAAATTTTGGCCACAGT	187089741	TGACACTGTCCATTCA	187090285	544	✓
2	+	TCTTCATACATTTTTTA	192291852	CTTCTACGCAGAGATT	192288818	3034	✓
2	+	CCTCATTACCTTCTGCC	217102433	ATGCCTCATTACCTTC	217102430	3	✓
2	-	CTGAACCCAGGCAGCTG	7788194	GTCCACGGCAGCGCTG	7788214	20	✓
2	-	GGATAGACCCAGAGCCG	64831325	CATGGCTGTCTGTCTG	64831451	126	
2	-	CCTTCCAGAGAAGACAC	73826930	CAGAAGTATGGAGGAG	73827068	138	✓
2	-	TTTACCTATACACTAAG	86101522	TGCTGCCTTCACCTTC	86100965	557	✓
2	-	AGTGAGGTGATTGAGAG	111670518	TGGGTTCCCGGACAAT	111670624	106	
2	-	CTTAAACTATCTTATTT	111677296	CCTCAAGAAAGAAAGC	111682100	4804	
2	-	TGCTTGAAATAGATTTA	206567828	ATTTTGCCTGGGGGAA	206568038	210	
3	+	TAGATTAACAAGTTAAT	45725283	TGTTTTTGCTCGGAGT	45724740	543	
3	+	GCATTCTTGTTTTACCC	109335654	AGTCTGATTGGTTGGA	109338122	2468	✓
3	-	CAAGGTTATTTTGCATC	14959677	TGGCAGTACCCAGCCC	14960080	403	
4	+	CCTGGCTAAAGTAATAT	39850875	CCTGGCAAGTTAGATG	39851380	505	
4	+	TAGGGGCTCACCAAACA	53274022	CTGTCTGGAGTCACAA	53274273	251	
4	-	TGTCCACACATTCATAA	81045787	CAAAGTCATGTGTCCA	81045797	10	
4	-	TGACAGCTTCTGCTAGG	90378940	ACTTTTGGTCAACCAC	90377906	1034	
5	+	TGCAGCAGAAAGACCTC	14752542	CTCCAGACCTGCAAAG	14752154	388	
5	+	CCTCACCAGGCCTGCTA	118722587	CCCTGTCGCCCTCTCT	118722939	352	✓
5	+	GGAGTGTGTGCCTGTAA	131470121	CTTGGGAGCCTCCAGG	131467033	3088	✓
5	+	GTTCTAAGTAAATGATT	141903661	AGGATTCCGCAAGCCT	141902249	1412	✓
5	+	TATGTGTTCTCTCTCCC	151170993	TGTGTCTGAGTCATCT	151171043	50	✓
5	+	TTTGTGTTACCATCGTG	175070321	CCCCCAGAGGTGCTTA	175069985	336	
5	-	GGGACAACCCAGTGACT	180189614	GGTCACACAAGCTGTT	180189798	184	
6	+	ACCCCACTTTCATTAAA	7226582	CGGGAAGCCATCAGTC	7227785	1203	
6	+	CGTCGAGCAGGACTATC	11243693	CGCTCGCCAAAGGAGG	11242766	927	✓
6	+	TCATACCTTTGTCAAAA	32977497	TCTCTACCCAAGTTGT	32977232	265	✓
6	+	TGTAGCTCTTCAGAAAG	45637271	TGGAAGCCTGAGTAAT	45637161	110	
6	+	AAACTGCAAGTGGTTCT	115429456	ATTGCCCAACTTCTCA	115429643	187	✓
6	+	GCAGAGACTAAAAATAT	149519375	GCCAACAGTCACTTGT	149519237	138	
6	-	TTTACAATATTTAGAAA	30366585	AGGACACGAGGAAGAG	30367483	898	
6	-	TGTAAATTTCATTCTTT	136000295	CCTGCTGCTTGTTGTT	136002609	2314	✓
7	+	TGCTATTGAGTACCTAA	22867401	ACTGTGGGCCTGGGCC	22866795	606	✓
7	+	AGATTGATCTCAAGGAG	127527214	TCAAGGAGTCAGTGGT	127527223	9	
7	+	TTTTTCCTTAATGATGT	135010111	TGACTTTTTTCCTTCT	135010136	25	
7	+	TTGTGCTAATTCCCTTT	148465721	CCTGGCTGTGCGTGGT	148468776	3055	✓
7	+	CTGCTGCCACCCCAGAC	149699582	CCAAGGCATTTACTCC	149701594	2012	
7	-	AGTAACTGTTCATTTTC	30523253	TGGCCTAATTCTCCGA	30521747	1506	
7	-	GTTTTGCACGTTTCGGT	44883556	AGGCTGGTTGGAAGAA	44884200	644	
7	-	CAGCCTGAGGCTCTTGG	126774158	TCCTGCTCCACGGAGC	126774394	236	✓
7	-	TAATGGACCCTTTCCAA	130237023	CTCCCGGCAACTCTGC	130237378	355	✓
7	-	ATTCTTGGGTGTATTAA	130245807	TCGTGGGCAAGGGCTG	130250498	4691	
7	-	AAATCGAGCATTTTTTT	130281927	GGTGAAATGAATTTCG	130281956	29	✓
7	-	ACGTCGTGTGCTTCTAA	138897225	ATTTTTTCGGTCTCCG	138896990	235	
7	-	GCAGTGCGGGAAGCTAA	154619274	TGGAGGAACACTTGGA	154618567	707	✓
8	+	AGTGACCCCAGACAAAC	8827860	GTTGCGGTCAACATGT	8827831	29	
8	+	GATCAGTACTAAAAGGC	79894272	TGTTTCTGGGCCATTT	79896854	2582	
8	+	AAGCTGTCTCCTGGCTT	128978396	CAGAAAGCTGCCACGG	128975865	2531	✓
8	+	GCTTAGGCCAGTGCTTA	129178139	ACCCCAGGAACGCTTG	129177991	148	
8	+	CCATTGAATGAGGGTAA	129634083	TGTCCTCAGTGTCTAC	129637078	2995	✓
8	-	AAGTTGCATCTGAGATG	71184924	AGACAGCTTGAATCAG	71187634	2710	
8	-	AATTTTTAATAAAACGT	101777196	ATTTTGCTGAGCTTGT	101777280	84	
8	-	TTCTAAGCTTTCTTCAG	141601738	TACCTCAGTTAAACAG	141601605	133	
9	+	CTGAAATGTTTAGGGGT	4883359	CATGCACCCACAGGAA	4882982	377	✓
9	+	TTCTATTGTTTTCCTTT	37079063	TCAAATGTGGGAAGGA	37079212	149	
9	+	TCTGCAAATTCTTGGAC	91524092	CTGCAGAGCCTCCCAT	91524362	270	✓
9	+	ACAACTGATCCAATTAC	92850925	CAATTACCCAGCCAGC	92850935	10	✓
9	-	AGCGATTCTAAGCCCAC	21314135	CGCCAGCCCAAACAGA	21314199	64	✓
9	-	TGTTCAAAGTGCTTGCT	37869455	TCGGTTGAAGTGTCAC	37869753	298	✓
9	-	AATTATACTTTTCAGGT	98695028	ATATTGTGGCAGAAGA	98695061	33	
9	-	CAACAGCCCATTATTGG	116695810	CTCCACCATTCACACT	116696252	442	
10	+	GAGGTCACTGTGAGGCT	97630135	TGGTCCATCTCCTGTA	97633242	3107	

Chromosome	Strand	SAGE		MPSS		Distance between tags	Masked region?
		Tag sequence	Start position	Tag sequence	Start position		
10	+	ATACGTATATTGTAATA	112272871	AGTGTGGTTTGGTGGT	112272330	541	✓
10	+	AATATATGAGCACTTTC	112276890	GCCCCACCAAGTAGGA	112280109	3219	✓
10	-	CCAAAACTTACCAAACT	6083615	AAACACTGTCAGGGGA	6083683	68	✓
10	-	TAACCAAAGTAAAAAGG	112224267	AGGACGGAATTCCAGA	112224910	643	
11	+	CCAGCAGCCCCTTTTTT	64947316	GGTGTTGCTTGCCTGC	64947892	576	
11	+	GATCTCAGCCTGCAGTG	85675844	TCAGCCTGCAGTGTGA	85675848	4	✓
11	+	GATTTACATATGATCAA	109487244	ATCCCGTGCTGTGATA	109486982	262	
11	-	TGGTGGCATACACCTGT	8650914	TGCAATGTCTAAGTTT	8647052	3862	✓
11	-	ATTTTAGTCATTTTAGT	58015767	TATAGCAGACTCTTCC	58018128	2361	
11	-	GATTACAGTTTTGACAA	60552830	GCTTGTGCCTAGGCAT	60552487	343	✓
11	-	ATTTTCAATCACAAGAC	64949736	CATTCGCTTTTGAAGG	64944926	4810	
11	-	AAAGTATGAAAGCAGCA	71261921	TGAAAAAACGGAGAGC	71262022	101	✓
11	-	GCAGCCGACCTCGGTTT	95551095	TGCCTGATACACAGTA	95555182	4087	✓
11	-	CAGAGCGGGCCTGCAGG	95622926	CAATTAGAGGGGGAAA	95621454	1472	✓
11	-	GTGATATATTAGAAATC	95634710	TGCCTCCTCTGCTGAA	95633652	1058	
12	+	AATGGCAAACAAACGGA	6864711	CTGAAGCCCAACTTCC	6863444	1267	
12	+	GTACTGTTGTAACCGTC	9692683	CAACTTGTTTTTCCAG	9692487	196	
12	+	AGAATCGGCTGAACCCA	19603531	CACTGAGGGGGATGCC	19603867	336	✓
12	+	TCCACGGAGCGTTTCTG	45937520	CAGCTCCCCGTTTTTA	45937540	20	
12	+	ACGAAAGAGCGAAACTC	48291881	AGTAGATGCCAGAAGC	48296739	4858	✓
12	+	GGAGGATAATATGCATT	67351900	CTGAGGAAGGGGAGGG	67356675	4775	✓
12	-	GAATAATTGTATTTTGC	24855676	ATCCGCAGGCTATGTT	24855870	194	
12	-	AGAGGTAGAATTTTTCT	91002814	CAGTGTGAGATACGTG	91004334	1520	
12	-	CTTGGGGACTGACAAAT	91029994	TGGCTGTCAAGACAAA	91032227	2233	✓
12	-	CACCTGGATACCTGCCA	125780411	ATTTCCTGTTCTCTCC	125780462	51	✓
14	-	ACAAGGATAATGCAGGA	99569061	CTAGCAGACAAATAGG	99571824	2763	
15	+	TTTGTTATTGTACCACA	56941234	TCTTTGTGCCAGGCCA	56940781	453	
15	+	TATTTGAAATTATTTCC	91233234	CACCTGGGGAGCCCAA	91235951	2717	
15	-	CATAACTTTGCACAGGA	47892419	AGGGCCTGGGACATTG	47891369	1050	✓
15	-	GGCTCATTTTGTGTCTA	47906788	ACTGGGGGTCATTTCA	47907421	633	✓
15	-	CAAGTTTGGAAACAGTA	58568267	GTCCAACTTTTCACTT	58568104	163	
15	-	GCTCCCTGGTGCCTAAA	99520183	AGCCCCTCCCAAGTTG	99520112	71	✓
16	+	ATCCCTGCACCTTTGCT	10388966	CCTGCACCTTTGCTTC	10388969	3	
16	+	CCTAAACTTTTGGGGGT	81237048	AGAAAGCGGAAGCAGA	81234514	2534	✓
16	-	TTATTTTTCTCTGAGTA	45388395	TCAGAACCTCTGTTCA	45388154	241	✓
16	-	TGATTCTATTAGGCAAT	56200724	TCAGCAATTTGGGGCT	56200969	245	✓
17	+	TGTCCAAGAATAATCCA	24901085	CTTCTCTGCTAGAACA	24901322	237	
17	+	AGGGCTGGATTTTTGTC	30326597	TCCCACACTAGTTTGC	30326575	22	✓
17	+	TAGAAGCAATTCTGGGG	31489444	AAGACAGAGTCTGAAG	31489003	441	
17	+	TTGTTTTTCTGTGTATC	53530209	ACCCCAGGGAATGTTG	53529953	256	✓
17	-	CTTGTTTAAGGAAAAGA	250339	TCTGAGAACCCCAGTC	251092	753	✓
17	-	AGGCCCCAAAGTGCCAA	10517017	TGTGTCTTGGTAGTGG	10516636	381	✓
17	-	TGGCTGTTACTAGGTTC	26120118	AAACCTAACATTACCT	26124172	4054	✓
17	-	ATTTGATTACTTTTTTC	34663636	CGAATAATGTGGCCTG	34663059	577	
17	-	TGTGGAACTGTGAAGTT	35167677	ATTTTGGCTACTGGAA	35167976	299	
17	-	GATGTGAAAAGCATTAA	46609883	ATTGAGAAAGTGTTTG	46609996	113	
18	+	TCACTGATTGCATTCCA	8622230	ACTGGGATGCGGTTCT	8626262	4032	✓
18	+	GAGCAGCAGCGGCGGCG	40513634	CCAGGCCACGAACTGT	40513210	424	✓
18	+	GAGGCATAAACAGAGGA	59776487	ATAGTTTGAAACACCC	59776844	357	✓
18	-	GACGGGTTGCCGAAGAG	12057318	CCTGGGCTCGGGCGAC	12057337	19	
19	+	CGTGAGATAAGAAATTT	46734261	ACCCTGGCCCACCATG	46734744	483	✓
19	-	CAAATTTCCAGCAAACC	6530825	ACCCGCCTCGGCTTGG	6531476	651	✓
19	-	GCAAGGCAAAAGTCCTC	32957755	AACTTCATCGTGCAGG	32960361	2606	
19	-	TGTTCTTGCACTGATGT	46734857	CCAGACGAGCCACCAG	46735458	601	✓
20	+	GTGTTAGAAGCCAGGGG	3802886	CATGGTGTTAGAAGCC	3802882	4	✓
20	+	ACTGGCTGCTGAATTCT	18498045	CTCAACTATTGGAAAT	18497998	47	
20	+	TAGTTGTATTTCTTGGG	30402962	CCATTAGACCAGGGGC	30402541	421	✓
20	+	TGGGAACTGCCTGTGTG	47646292	CCAAGGGAGGCAGTGC	47644602	1690	
21	+	GAAAATTTGTAACCCTC	25858375	TTACTGGACTCAAAAC	25859261	886	
22	+	GGGCTGGGCCCTGGACA	44815021	TGAATGGCCGGTGTGT	44811671	3350	
X	+	CAAAATCCAATAGCAAC	2566157	TTCACATTCAGGTTTC	2566234	77	
X	+	GGACCAAGAGGGACAAC	53762199	AGCGAGAGTACAGAAG	53759213	2986	
X	-	GGCCTGAGCCCCAGTTG	39050080	CATGCAGCCTCTGAGA	39050107	27	✓
X	-	CAAAAACACATTGATAA	62563653	AACGACGCCCTTTTTC	62563521	132	✓
X	-	GGTGACCACCAGAAAGT	72957319	ATTGTTGAGATGAGGA	72958118	799	
X	-	CGTTGTAGAGTGGGAAT	72959386	ACCTTTGACATGCGTT	72959398	12	
X	-	GAAACTGCTTTTAAACT	72961765	CAAGACCAAAACAAGG	72963741	1976	

Table S2: *Novel loci of transcription identified by combining LongSAGE and MPSS.*
Each loci listed is defined by both a LongSAGE tag and an MPSS tag that match the genome uniquely at positions within 5000 bases of each other and more than 5000 bases from any known exon in the Ensembl database.
